# A descriptive analysis of relations between parents' self-reported smoking behavior and infants' daily exposure to environmental tobacco smoke

**DOI:** 10.1186/1471-2458-10-424

**Published:** 2010-07-19

**Authors:** Doris Kehl, Jochen R Thyrian, Jan Lüdemann, Matthias Nauck, Ulrich John

**Affiliations:** 1 Ernst-Moritz-Arndt-University Greifswald, Institute of Psychology, Department Health and Prevention, Robert-Blum-Str. 13, D-17487 Greifswald, Germany; 2 Ernst-Moritz-Arndt-University Greifswald, Institute of Community Medicine, Section Epidemiology of Health Care and Community Health, Ellernholzstr. 1-2, D-17487 Greifswald, Germany; 3 University Hospital Greifswald, AoR, Institute of Clinical Chemistry and Laboratory Medicine, Sauerbruchstrasse, D-17475 Greifswald, Germany; 4 Ernst-Moritz-Arndt-University Greifswald, Institute of Epidemiology and Social Medicine, Walther-Rathenau-Str. 48, D-17487 Greifswald, Germany

## Abstract

**Background:**

The aims of the present study were to examine relations between parents' self-reported smoking behavior and infants' daily exposure to environmental tobacco smoke, as assessed by urinary cotinine-to-creatinine ratio (CCR), and to describe the CCR over seven days among infants at home.

**Methods:**

A convenience sample of 27 households was drawn. Each household had to have at least one daily tobacco smoker and one child up to three years of age. Over a seven-day period, urine samples were obtained from the child daily. To examine relations between parents' self-reported smoking and infants' daily CCR, generalized estimating equation (GEE) analysis was used.

**Results:**

The data revealed that infants from households with indoor smoking had higher CCRs than infants in households with outdoor smoking. CCRs were higher in girls than in boys. Older infants had lower CCRs than younger infants. Smoking outside the home versus inside the home, infant's gender, and infants' age accounted for 68% of the variance in CCR in a GEE data analysis model. No increase or decrease of CCR over time was found.

**Conclusion:**

The findings suggest that parents' self-reported smoking indoors at home versus outdoors is predictive of CCR among infants three and younger. Higher CCR concentrations in girls' urine need further examination. Furthermore, significant fluctuations in daily CCR were not apparent in infants over a seven-day time period.

## Background

Exposure to environmental tobacco smoke (ETS) is an important health risk among infants [1]. Infants lack mobility and independence and are unable to complain about or avoid ETS [2]. In homes where tobacco smoking occurs, infants are exposed during their regular daily activities [3]. Many studies have shown strong and consistent associations between ETS, particularly from parental smoking, and numerous childhood diseases such as respiratory infection, asthma, middle ear infections, and sudden infant death syndrome.

Infant's exposure to ETS at home may be expected to differ according to the amount of tobacco smoke and the distance of tobacco smoke in relation to the air that the child breathes. Research suggests less exposure to ETS in homes with a home smoking ban than in homes without such a ban [4,5]. However, children are exposed to ETS across different environments [6,7]. Parents may not be aware of ETS exposure that occurs outside the home [8].

Cotinine has been suggested as the biomarker of choice for measuring exposure to ETS [9]. Cotinine may be taken from different tissues or fluids in the body [10]. Among infants at age three or younger, urine samples seem to be particularly feasible since blood samples are more invasive and hair samples may not be available or may not be tolerated by children or parents. Cotinine may indicate exposure to ETS over the last 24 hours due to its half-life of approximately 20 hours [11]. By the use of cotinine-to-creatinine ratio (CCR) dilution of the urine may be considered [12].

There is little research about the relationship between smokers' self-reports of smoking indoors and their childrens' exposure to tobacco smoke. Children in households with home smoking bans had lower cotinine than those in homes without a smoking ban [5]. Additionally, smoking and the number of cigarettes per day (cpd) smoked in child's presence were associated with urinary cotinine [13]. Among children exposed to more than 10 cpd, the median CCR was 2.4 times higher than among those unexposed to smoke [14]. Children in households with one or more current daily cigarette smokers, compared to their counterparts in households without cigarette smokers, were more likely to have a urinary CCR greater than 32 ng/mg [15]. In addition, consistency and variability of CCR has been assessed every other month from birth to two years of age and how well self reported ETS data predict multiple CCR measurements was analyzed [15]. The findings revealed that variation in ETS exposure over time is reflected in the CCR values. This raises the question whether the CCRs fluctuate if daily measurements, including weekends, are taken. It also raised the question of whether daily measured CCRs are related with parents' reports of smoking.

Limitations of the current research are that CCR has not been taken daily over one week, including a weekend, or longer. Hence, it seems to be unknown whether the CCRs vary if daily measurements are taken. The aim of the present study was to examine relationship between infants' daily exposures to ETS, as assessed by urinary CCR, and parents' self-reported smoking over a seven day period. We also wanted to describe CCR over seven days among infants' at age three years or younger.

## Methods

### Sample

A convenience sample of 27 households in Northern Germany was drawn. Households could be included in the study if they had a child aged three years old or younger and if there was at least one daily smoker present at home, i. e. a person that had smoked at least one cigarette per day for the last four weeks prior to the study [16]. Average age of the 28 study infants was 18.04 (*SD *= 12.07) months, with a range from 2 to 43 months. During the study period, only one of the children was breast-fed, and this child was breast-fed by a mother who did not smoke at the time of study. Half of the infants were girls. In one household lived twins. Among the households, 22 were recruited proactively in one pediatrician's office, and five were recruited through advertising in pediatricians' offices, in a newspaper or through friends. Informed written consent was obtained from all parents of the children prior to their participation in the study. Participants were offered 30 Euro that they received at the end of the data collection. Data were gathered in the city and suburbs of Rostock, Germany.

The study was part of the project about reduction of ETS in families and was conformed to the principles of the Helsinki Declaration as reflected by an a priori approval of the Ethics committee of the University of Greifswald (project GESA: reference number BB 64/07).

### Assessments

Parents took part in a 10- to 15-minute interview about their smoking status, household smoking habits (including smoking on the balcony or outside, instead of inside the home) and socio demographic characteristics. The interview took place in the respondents' homes. Parents' age, sex, native language and presence of a partner were recorded. Level of education was grouped into the following categories: less than or equal to 9 years, 10 to 11 years, and 12 to 13 years of school. Employment status was classified as "employed," "unemployed," "parenting time," and "other."

The smoking status of the adult respondents was recorded. Individuals were classified as ever smokers if they smoked more than 100 cigarettes in their lifetime. They were classified as current daily smokers if they had smoked one or more cpd throughout the last 30 days. Parents who smoked during the last 30 days but not daily were classified as less than daily smoker. Former smokers were those who had smoked more than 100 cigarettes in their lifetime but did not smoke during the last 30 days. Mean cpd was obtained from current daily smokers. Additionally, respondents were asked whether they had smoked tobacco other than cigarettes. Smoking status was determined for each adult who lived in the household. Furthermore, participants were asked whether the smoking occurred in the home, and if so, in what rooms. Participants were also asked if they smoked in the living room during the seven days prior to the interview. Additionally, the infant's sex and age (in months) were recorded, as well as whether the infant was breast-fed.

For the study period of seven days, participants had to complete a "smoking diary" that included the following information: cpd smoked in the presence of the infant at home, on the balcony, in the car or outside home. Presence was defined as the possibility for the smoke to reach the child via air flow. If parents smoked outside the room where the child was, with the doors closed, this was considered not smoking in the presence of the infant, even though particles of tobacco smoke likely existed in the parents' clothes. For the study period of seven days, parents were also asked if their infant spend time (in the daytime or in the evening) in rooms in which smoking occurs, and if yes, how many minutes.

Nicotine exposure was assessed for seven consecutive days by nicotine metabolite monitoring (mainly cotinine) from daily urine samples from each child and parent. Parents' urine was collected in a vacuum blood collection tube (Vacutainer^®^). If infants were toilet-trained, urine was collected in a vacuum blood collection tube or was absorbed with a cotton roll. Among infants who were not toilet-trained, the cotton roll was placed into the diaper to collect infants' urine. If wet, it was returned to the hygienic vial (Salivette^®^, Sarstedt, Nümbrecht, Germany). Urine samples were stored at -20°C until analysis. Samples were analyzed for quantitative determinations of cotinine and other principal metabolites of nicotine on an Immulite 2500 analyzer via a solid-phase competitive chemiluminescent immunoassay (reagents and analyzer from Siemens Healthcare Diagnostics, Frankfurt, Germany). The reportable range was between 10 and 500 ng/ml, and the detection limit for cotinine was 10 ng/ml. Analytical sensitivity is 5 ng/ml. Urine creatinine measurements were performed photometrically on a Dimension RxL (Siemens Healthcare Diagnostics, Frankfurt, Germany). To adjust for dilution of the urine, CCR were calculated [12]. CCR was expressed as μg of cotinine per mmol of creatinine.

### Data analysis

Cotinine values greater than or equal to 500 ng/ml were used to distinguish tobacco smokers from non-smokers or from passive smokers only. To interpret the cotinine values of passive smokers the following categories of exposure were used: low exposure (10 to14 ng/ml), moderate exposure (15 to 40 ng/ml), and heavy exposure (more than 40 ng/ml) [17].

Because the average of the multiple measurements should provide the best available estimation of ETS exposure [15], the median and the mean with standard deviation of cotinine and CCR for each infant over the study period was calculated. The intra-individual means and standard deviations were then averaged across all infants. Because the reportable range of the analyzer was 10 to 500 ng/ml, cotinine values less than 10 ng/ml were assumed to be 10 ng/ml for the calculation of descriptive statistics. All seven urine samples were obtained from 25 of the participating infants. For one infant, none of the seven urine samples could be analyzed due to insufficient urine. Among all urine samples collected (191 out of 196), 13.1% could not be analyzed for cotinine and CCR due to insufficient urine.

Generalized estimating equation (GEE) was used to determine how the self-reported smoking behavior of adults is related with childrens' urinary CCR over time. The strength of the relation between the childrens' daily urinary CCR and the ETS exposure variables can best be determined by using GEE analysis [18]. Accurate inferences can only be determined by taking into account the within-participant correlation between repeated measurements [18]. Cotinine values of less than 10 ng/ml were set to zero before the CCRs were calculated. As the CCRs were not normally distributed, a logarithmic e-transformation was performed. The longitudinal design involved seven repeated measurements of infants' urinary CCR _log e_. Within-infant correlation of these values was expected. Predictor variables included smoking outside the home (compared to inside the home), one or two smokers at home, cpd smoked in the presence of the infant at home, on the balcony, in the car or outside home, and time infants spend (in the daytime or in the evening) in rooms in which smoking occurs. Due to potential multicollinearity - the fact that independent variables should not be interrelated - each predictor variable was first entered separately into a GEE analysis. These predictor variables were adjusted for study days, one through seven, for the assessment of the within-participant correlation between repeated measurements. The significant predictor variable was again entered into a GEE analysis that was adjusted for study day, infants' age (in months) and infants' sex. Finally, the explained variance of the models was calculated as follows: 1-(scale parameter^2^/standard deviation of the outcome variable^2^) [19]. Pearson correlation coefficients of CCR _log e _over the seven-day study period were calculated. The coefficients were required to assess the variability of CCR _log e _over the seven-day study period, respectively, over the weekdays, including all infants. Furthermore, an intra-class coefficient (Two-Way Mixed Model, Type Absolute Agreement, Single Measures ICC) was calculated over the study period. Analyses were performed using Stata 9.

## Results

Demographic and smoking characteristics of the adult respondents are provided in Table 1. There was no significant relation between the age of the mother and the infants' mean cotinine value, τ = .23, p = .11. There was no significant effect of mothers' school education on infants' mean cotinine levels, *F *(2, 24) = 2.52, *p *= .10, *ω *= .10, although a small effect was observed. Additionally there was no significant effect of household income, *F *(2, 24) = 1.08, *p *= .36, *ω *= .01, and employment status of the mother, *F *(3, 23) = .58, *p *= .64, *ω *= -.05, on infants' mean cotinine levels.

**Table 1:** Demographic and smoking characteristics of the adult respondents (N = 47).

	N (%)
**Demographic characteristics**	
Age	
*Mean *27.47	
*SD *5.32	
Female gender	27 (57.45)
Partner present	42 (89.4)
Native language German	39 (83.0)
School education	
Low (≤ 9 years)	16 (34.1)
Middle (10 - 11 years)	19 (40.4)
High (≥ 12 years)	12 (25.5)
Employment	
Employed	16 (34.0)
Unemployed	9 (19.1)
Parenting time	16 (34.0)
Other	6 (12.8)
Household income per month ^a^	
< 1300 €	14 (53.9)
1300 - 2300 €	8 (30.8)
> 2300 €	4 (15.4)
**Smoking characteristics**	
Smoking status of respondent ^b^	
Daily smoker	39 (83.0)
Less than daily smoker	3 (6.4)
Former smoker	2 (4.3)
Never smoker	3 (6.4)
Cigarettes per day (no.), daily smokers (n = 39)	
*Mean *16.23	
*SD *7.25	

^a ^one household income is missing ^b ^daily smoker: smoked more than 100 cigarettes in lifetime and currently smoking at least one cigarette daily over the last four weeks; less than daily smoker: smoked more than 100 cigarettes and less than daily during the last 30 days; former smoker: smoked more than 100 cigarettes in lifetime and did not smoke during the last 30 days prior to the interview; never smoker: did not smoke, or smoked less than 100 cigarettes in their life.

Daily smokers' cotinine concentrations during the study period of seven days were 500.0 or more ng/ml. The mean cotinine concentration among former and never smokers was 40.0 ng/ml (*SD *= 36.6). Consequently, self-reported smoking status was related with cotinine values.

Among all households, 14 included two smokers and 13 included one smoker, and in 14 households there was smoking inside the home. Among the 14 households with indoor smoking, in 10 tobacco smoking took place in one room only. This room was the kitchen in eight households, the living room in one household, and the working room in one household. In four households, there was general tobacco smoking in two rooms (the kitchen and one of the other rooms such as the bathroom, housekeeping room or living room). No smoking in the bedroom or the children's room was reported. For the seven days prior to the interview, there was smoking in the living room in 6 of the 14 indoor smoking households. In these 6 households, it was one household member (with or without visitors) who smoked.

The median of the cotinine concentrations across all infants was 28.2, concentrations ranged from less than 10.0 to 179.0 ng/ml. Six infants had a median less than 10.0 ng/ml, 2 had a median between 10.0 and 14 ng/ml, 9 a median between 15.0 and 40 ng/ml, and 10 a median above 40 ng/ml. The mean cotinine concentration across all infants was 52.7 (*SD *= 47.7), and it ranged from less than 10.0 to 171.4 ng/ml. Three infants had mean cotinine values less than 10 ng/ml, five had a low mean (10-14 ng/ml), seven had a moderate mean (15-40 ng/ml), and 12 had a high mean exposure to ETS (more than 40 ng/ml). For one infant, none of the seven urine samples could be analyzed due to insufficient urine. Among the 14 infants who had reports of smoking inside the home, all had mean cotinine concentrations greater than or equal to 10 ng/ml. Among the 13 infants who had no reports of parent's smoking inside the home, nine infants had mean cotinine concentrations greater than 10 ng/ml and three infants had mean cotinine concentrations less than 10 ng/ml. From Monday to Sunday, the mean cotinine concentrations across all infants ranged from 31.8 (*SD *= 31.4; Thursday) to 61.5 (*SD *= 77.0; Wednesday). The mean CCR across all infants was 43.2 (*SD *= 96.3), and ranged from 0.43 to 512.7 μg/mmol. From Monday to Sunday, the mean CCR across all infants ranged from 24.7 (*SD *= 33.0; Monday) to 63.9 μg/mmol (*SD *= 111.7; Sunday). Infants' cotinine creatinine ratios (microgram/mmol) in urine during the period of 7 days are presented in Table 2.

**Table 2:** Infants' cotinine creatinine ratios (microgram/mmol) in urine during the period of 7 days

Subject no.	Sex	Age (month)	Cotinine creatinine ratio (microgram/mmol) in infants' urine									Indoor - outdoor smoking	No. of smokers in home
					
			mon	tue	wed	thu	fri	sat	sun	mean	sd		
1	F	2	165,93	664,62	570,83	937,50	290,00	470,67	489,23	512,68	251,36	indoor	2
2	F	3	20,41	20,83	22,34	38,46	22,73	*	17,86	23,77	7,40	outdoor	2
3	F	4	*	*	*	*	*	*	*	-	-	outdoor	1
4	F	6	11,64	20,37	22,05	14,04	14,77	12,30	18,57	16,25	4,08	outdoor	2
5	F	6	5,71	6,17	16,95	13,51	10,99	13,33	8,26	10,71	4,19	outdoor	1
6	M	6	8,95	6,91	8,70	5,45	-	-	-	7,50	1,64	outdoor	1
7	F	7	42,15	87,80	42,86	*	*	80,88	175,74	85,89	54,47	outdoor	1
8	M	8	32,70	44,35	36,59	23,28	26,44	29,68	25,66	31,24	7,33	indoor	2
9	F	8	39,76	41,57	32,63	29,89	112,17	77,33	157,14	70,07	48,57	indoor	2
10	M	8	16,67	10,05	13,41	17,81	18,98	*	*	15,38	3,63	indoor	2
11	F	11	33,33	24,20	80,41	*	26,53	*	31,06	39,11	23,37	indoor	2
12	M	11	8,10	13,21	22,18	25,40	125,00	20,33	6,08	31,47	41,87	indoor	1
13	F	12	18,52	32,31	32,66	85,29	40,22	8,22	200,00	59,60	66,51	indoor	2
14	M	13	1,31	3,48	5,62	2,62	2,13	1,98	3,52	2,95	1,42	outdoor	1
15	M	18	2,94	5,00	3,50	2,82	22,73	2,26	3,83	6,15	7,36	outdoor	1
16	F	20	65,86	74,55	18,87	40,23	41,11	87,80	96,30	60,67	28,20	indoor	1
17	M	20	*	*	3,68	*	*	3,08	*.	3,38	0,43	outdoor	1
18	M	23	12,94	11,98	12,89	20,00	16,60	42,36	17,41	19,17	10,63	indoor	1
19	M	26	4,95	2,53	3,30	1,92	24,39	3,38	3,45	6,27	8,04	outdoor	2
20	F	26	23,61	20,38	27,21	*	*	*	*	23,73	3,41	indoor	1
21	F	27	8,17	3,70	15,15	6,02	16,65	5,84	7,90	9,06	4,92	outdoor	2
22	F	29	5,88	2,01	2,89	2,77	10,10	2,24	4,39	4,32	2,89	outdoor	2
23	M	30	6,67	6,27	4,60	4,81	10,96	13,05	11,67	8,29	3,50	outdoor	2
24	F	32	27,04	43,35	26,49	-	31,72	31,15	38,02	32,96	6,56	indoor	1
25	M	33	14,85	8,96	7,54	-	24,79	13,76	*	13,98	6,79	indoor	1
26	M	35	*	20,88	15,38	18,12	37,14	46,23	40,00	29,63	13,05	indoor	2
27	M	38	17,23	18,99	19,50	15,06	13,12	15,64	14,18	16,25	2,41	indoor	2
28	M	43	21,64	28,25	26,12	22,99	18,05	34,06	36,23	26,76	6,61	indoor	2

Mean (SD)			24.68 (33.01)	47.03 (127.79)	40.53 (107.23)	63.24 (201.21)	41.62 (61.71)	46.16 (98.39)	63.93 (111.65)	43.23 (96.27)			

Notes: - = no material; * = too little material to analyse urine.

Relations of the infant's daily CCR with ETS exposure variables are presented in Table 3. An exchangeable working correlation structure was used regarding the calculated correlation matrix. Smoking outside the home, compared to inside the home, was the only exposure variable that was significantly related to CCR. Infants in households with indoor smoking had significantly higher CCRs than infants in households with reports of smoking on the balcony or outside the home. The explained variance of this model was 22%. For infants with one smoker in the home, compared to infants with two smokers in the home, the differences between the CCRs were not significant. Cpd smoked in the presence of the infant and time infants spend in rooms in which smoking occurs were not significantly related with the CCR.

**Table 3:** Smoking at home and infant cotinine-to-creatinine ratio

	Cotinine-to-creatinine ratio (μg/mmol) with log _e _transformation					
	
	*B*	*SE*	*z*	*p *> |*z*|	95% *CI*	
Household smoking						
Smoking outside the home	Ref.					
Smoking inside the home	1.23	0.41	3.00	< .01	.43	2.04
Number of smokers living in home						
One smoker	Ref.					
Two smokers	.33	0.44	0.75	.46	-.54	1.20
Cpd smoked in the presence of the infant	.02	0.05	0.43	.67	-.08	.13
Time infants spend in rooms in which smoking occurs (minutes)	.00	0.00	0.96	.34	-.00	.01

Generalized estimation equation models predict the infant's cotinine-to-creatinine ratio (CCR), taking into account cluster correlation within subjects across study days. Models are based on an exchangeable correlation structure for repeated observations within subjects. Predictor variables were adjusted for study day

Smoking inside versus outside home was again entered into a GEE model, and this variable included adjustments for infant's age (in months), gender and study day (Table 4). Smoking inside compared to outside home, as well as gender and age, were significantly related to the CCR. The CCRs were higher in girls than in boys. Also, older infants had lower CCR. The data also revealed that there was no significant increase of CCR over time. The final GEE model accounted for 68% of the variance in the CCR.

**Table 4:** Household smoking, adjusted for infants' sex and age and for study day, and infant cotinine-to-creatinine ratio

	Cotinine-to-creatinine ratio (μg/mmol) with log _e _transformation					
	
	*B*	*SE*	*z*	*p *> |*z*|	95% *CI*	
Household smoking						
Smoking outside the home	Ref.					
Smoking inside the home	1.42	0.33	4.32	< .001	.77	2.07
Infant's sex and age						
Male	Ref.					
Female	.79	0.26	3.01	< .01	.28	1.31
Age (month)	-.03	0.01	-2.02	< .05	-.06	-.00
Study day	.03	0.03	1.02	.31	-.03	.08

Generalized estimation equation model to predict the infants' cotinine-to-creatinine ratio (CCR), taking into account cluster correlation within subjects across study days. Model is based on an exchangeable correlation structure for repeated observations within subjects. Predictor variable household smoking was adjusted for infants' age and sex and for study day. Wald chi2 (4) = 40.53, scale parameter = .64. Explained variance of the GEE model was 67.5 percent.

To assess the variability of CCR of all infants over the seven-day study period, correlation coefficients were calculated. All 21 coefficients in the correlation matrix, for study day one through seven, ranged between .61 (3/7 day) and .96 (1/6 day) and were significant at a *p*-value of ≤.001; only one had a *p*-value of < .05. In the correlation matrix for Monday to Sunday, all 21 coefficients ranged between .69 (Wed/Sun and Thu/Sat) to .95 (Fri/Sun) and were significant at a *p*- level of ≤.001. On weekends vs. weekdays was no absolute difference of CCR. The intra-class-correlation over the seven-day study period was .88, *p *<.001.

## Discussion

There are three main findings of this study. First, smoking outside the home, compared to inside the home, was related with CCR. Second, female and younger infants revealed higher CCRs than their male and older counterparts. Third, significant fluctuations in daily CCR were not apparent in infants over a seven day time period.

Infants from homes with indoor smoking had higher CCRs than infants from homes where smoking occurred on the balcony or in other places outside the home. The data support preexisting cross-sectional findings that suggest a home smoking ban may reduce infants' cotinine levels [4,5]. According to dose-response relationships, the present data are somewhat contradictory. The cross-sectional data clearly revealed a dose-response relationship, but the longitudinal data did not. The mean CCR was higher (57.9 μg/mmol) in infants with two smokers in the home compared to infants with one smoker in the home (24.9 μg/mmol). This confirms findings from the German Environmental Survey IV that also revealed a higher geometrical mean of cotinine among infants in households with more than one smoker than in households with only one smoker [20]. In another study urine cotinine levels of infants' aged 3 to 27 month were correlated with the number of smokers in the home, however in this study also households with no smokers were included [4]. Our cross-sectional data clearly revealed a significant dose-response relationship between CCR and time infants spend in rooms in which smoking occurs in respect to study day 1, *r_s _*= .46, *p *= .01 (one-tailed), however our longitudinal data did not. Furthermore a positive but not significant relationship was revealed between CCR and cpd parents smoked in the presence of the infant in respect to study day 1, *r_s _*= .32, *p *= .07 (one-tailed).

Second, the present data revealed that sex and age of the infant predicted CCR. When these two predictors were included, in addition to smoking inside home, 68% of the CCR variance was explained. Only 22% of the variance was explained when only the information about smoking inside the home was used. CCRs were greater among girls than among boys. Mean creatinine was lower in girls than in boys. Whether total creatinine excretion over a 24 hours period was lower in girls is unknown. This would have required 24 hour urine volume measurements. The mean cotinine value tended to be higher in girls than in boys. This confirms former evidence about higher mean cotinine values in girls compared to boys [13].

Our data revealed that mean cotinine and CCRs were higher and mean creatinine values were lower in younger than in older infants. This is in accordance with the expectation that the concentration of the same amount of tobacco smoke is higher among younger than among older infants due to less body tissue of the younger [21-23]. We do not know, whether total creatinine excretion over a 24 hours period was lower in younger than in older infants. However, our finding according to which mean cotinine was greater in younger than in older children is consistent with previous evidence. It also revealed that younger children had higher urinary cotinine levels than older children [24]. Urinary elimination half-life of cotinine seems to depend on age [23]. However, elimination half-life of cotinine did not differ between infants and children [25]. A further explanation for higher cotine levels among younger than among older children might be that younger infants have a higher ventilation rate than older children or adults [23,24]. Parents might tend to spend more time with younger than with older infants. Furthermore, the greater lack of independent mobility and inability to detract themselves from exposure to ETS among younger infants could be a reason for higher urinary cotinine respectively CCR compared to older infants.

Third, the present data revealed that daily exposure to ETS, as assessed by CCR, was stable over seven days in the total sample. No absolute difference of CCR was found between weekends and weekdays. This finding suggests that weekends, or other peak smoking days, may not distort the overall findings. Instead, mean CCR over seven consecutive days, seems to be justified. The present data suggest that a single day measurement of cotinine in urine accurately reflects infant's exposure to ETS over one week period assuming that ETS exposure is consistent over that period. The finding may not be generalized to longer periods of time. A series of CCR assessments taken from infants during the first two years of their lives revealed variability over these two years [15]. Maturing of the infant and potential changes in exposure to smoke may explain this finding.

The present study has several limitations. First, the sample was a convenience sample. However, the number of cpd among daily smokers was in accordance with other studies [5,25]. Second, data about smoking and about ETS exposure of infants might have been biased by parents' self-reports, even though we have found CCR in the infants. Also, the sample size was large enough to test relations between parents' self-reports of smoking and ETS in infants. Third, adjustment of urine cotinine concentration by creatinine concentration in urine of infants is problematic because creatinine levels and creatinine excretion were lower in girls than in boys and lower in younger than in older children. Despite this fact, the cross-sectional data revealed that the mean cotinine values were higher in younger infants and tended to be higher in girls. Finally, the used immunoassay measures metabolites of nicotine, in particular cotinine, to which it is highly specific. Cross-reactivity between cotinine and other cotinine metabolites could occur. Since the assay is used for screening purposes rather than for determining cotinine concentrations per se, it also measures other nicotine metabolites. Assessing cotinine specifically, e.g. by high-performance liquid chromatography (HPLC), may give lower results. Nevertheless, we estimated the used immunoassay to be highly adequate to examine the longitudinal relationship between infants' exposure to environmental tobacco smoke and parents' self-reported smoking behavior.

## Conclusion

Smoking inside the home, together with the infant's sex and age, is crucial in predicting CCR in infants. The finding that CCR was higher in girls than in boys needs further examination. The data suggest that significant fluctuations in daily CCR were not apparent. Measurement of CCR in urine on a single day might accurately reflect infants' exposure to ETS over one week. The findings provide evidence about CCR for purposes of public health. The data speak for home smoking bans. In addition, the findings support routine cotinine measurement among children of smokers for purposes of feedback that might help to generate intention to quit smoking.

## Competing interests

The authors declare that they have no competing interests.

## Authors' contributions

DK, JRT and UJ developed the research question and designed the study. DK conducted the literature research. DK and JRT collected data. MN and JL were responsible for the cotinine and creatinine analysis and their interpretation. DK carried out the statistical data analysis and their interpretation and was responsible for drafting the manuscript.

All authors made substantial intellectual contributions to the work and participated in writing the manuscript by reviewing the drafts. All authors have approved the final manuscript.

## Pre-publication history

The pre-publication history for this paper can be accessed here:

http://www.biomedcentral.com/1471-2458/10/424/prepub
